# Composite Indium Tin Oxide Nanofibers with Embedded Hematite Nanoparticles for Photoelectrochemical Water Splitting

**DOI:** 10.1021/acsami.2c05424

**Published:** 2022-09-12

**Authors:** Oren Elishav, David Stone, Anton Tsyganok, Swetha Jayanthi, David S. Ellis, Tamir Yeshurun, Itzhak I. Maor, Adar Levi, Vadim Beilin, Gennady E. Shter, Roie Yerushalmi, Avner Rothschild, Uri Banin, Gideon S. Grader

**Affiliations:** † The Nancy & Stephen Grand Technion Energy Program (GTEP), Technion−Israel Institute of Technology, Haifa 3200002, Israel; ‡ Institute of Chemistry and the Center for Nanoscience and Nanotechnology, The Hebrew University of Jerusalem, 91904 Jerusalem, Israel; § Department of Materials Science and Engineering, Technion−Israel Institute of Technology, Haifa 3200002, Israel; ∥ The Wolfson Department of Chemical Engineering, Technion−Israel Institute of Technology, Haifa 3200003 Israel; ⊥ Faculty of Engineering, Tel Aviv University, Tel Aviv 6997801, Israel

**Keywords:** hematite, nanofibers, nanoparticles, photochemistry, water splitting

## Abstract

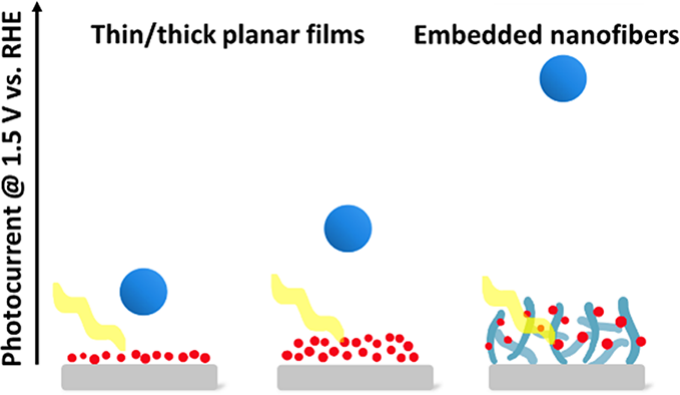

Hematite is a classical photoanode material for photoelectrochemical water splitting due to its stability, performance, and low cost. However, the effect of particle size is still a question due to the charge transfer to the electrodes. In this work, we addressed this subject by the fabrication of a photoelectrode with hematite nanoparticles embedded in close contact with the electrode substrate. The nanoparticles were synthesized by a solvothermal method and colloidal stabilization with charged hydroxide molecules, and we were able to further use them to prepare electrodes for water photo-oxidation. Hematite nanoparticles were embedded within electrospun tin-doped indium oxide nanofibers. The fibrous layer acted as a current collector scaffold for the nanoparticles, supporting the effective transport of charge carriers. This method allows better contact of the nanoparticles with the substrate, and also, the fibrous scaffold increases the optical density of the photoelectrode. Electrodes based on nanofibers with embedded nanoparticles display significantly enhanced photoelectrochemical performance compared to their flat nanoparticle-based layer counterparts. This nanofiber architecture increases the photocurrent density and photon-to-current internal conversion efficiency by factors of 2 and 10, respectively.

## 1 Introduction

Photoelectrochemical (PEC) water splitting is a promising route to store solar energy in chemical bonds by producing green hydrogen from water.^1−4^ Green hydrogen can facilitate the integration of renewable energies into the grid^5−7^ or be converted to other valuable chemicals.^8−10^ A key challenge for this route is to develop effective photoelectrodes for producing hydrogen at a competitive cost. Hematite (α-Fe_2_O_3_) is a well-studied photoanode material for PEC cells, given its stability in alkaline electrolytes, favorable band gap energy, high abundance, nontoxicity, and low cost.^11−13^ However, significant bulk recombination leads to insufficient charge carrier collection efficiency due to a very short diffusion length of 2–4 nm, hindering its practical implementation.^14,15^ Therefore, hematite-based nanostructured photoanodes were suggested to mitigate these limitations and improve performance.^16−18^ However, there are two challenges in such systems. First, while bulk hematite is a very stable phase of iron oxide, achieving control over nanosized hematite photoanodes remains a challenge, and in most cases, the particle size is still much larger compared to the hole diffusion length.^19,20^ In recent years, hematite particles as small as 20–30 nm were achieved through colloidal synthesis.^21^ The second challenge is to form a close contact between the hematite particles and the substrate electrode, to enable efficient charge separation and reduce series resistance losses. Despite much research on nanostructured hematite, the use of size-controlled NPs with well-defined shapes and exposed facets to fabricate photoelectrodes for PEC water splitting is still rarely reported.^22^

In addition to the design of the hematite itself, another complementary strategy is to control the structure of the transparent current collector layer that collects the (photo)current from the photocatalytic layer.^23^ Thus, systematic design of a nanostructured transparent conductive oxide (TCO) can enhance the photoanode performance. Specifically, Eftekharinia et al. performed full-field electromagnetic calculations, and long (1500 nm) TCO nanorods were predicted to achieve a higher photocurrent.^24^ As previously shown, one-dimensional (1D) nanostructured materials, such as nanowires and nanofibers, can significantly increase the number of reaction sites while still allowing effective charge separation.^25−29^ These structures improve nanoparticle integration while facilitating effective current collection.^30,31^ Therefore, such an architecture could increase the overall photoelectrode performance. For example, Lin et al. used TiSi_2_ nanofibers with a diameter below 100 nm as a scaffold for atomic layer deposition (ALD) of hematite. The scaffold provided structural support and effective charge transport with improved performance relative to the planar architecture.^32^ Similarly, transparent current collectors based on porous conducting nanofibers can be used as a scaffold.^33^ In addition, the architecture of the 1D array of TCO can allow geometric light trapping and enhance the photoanode performance. Suter et al. performed numerical calculations of the electromagnetic wave propagation for planar and ordered wedge microstructure architectures of an α-Fe_2_O_3_ photoanode.^23^ Resonant and geometric light trapping also increases the photocurrent density.^34^ Still, due to the trade-off between optical and electrical performance, a random nanostructure can achieve comparable performance to an ordered architecture.^35^

One of the most well-known TCO is tin-doped indium oxide (ITO), a useful material extensively utilized in the electronic and display industries.^36−39^ 1D nanostructured ITO can be utilized as a conductive scaffold for photoelectrodes. For example, Han et al. grew an ITO nanowire array by a vapor–liquid–solid method on commercial ITO-coated glass and deposited ALD layers of CdSe/CdS and TiO_2_. The nanowires array layer had an average thickness of 12 μm and generated a 2.4 times higher photocurrent than a porous film analog.^40^ In addition, previous studies examined the integration of photocatalytic NPs into a 1D nanostructured scaffold. Wei et al. decorated Au NPs on ZnO/CdS nanotube arrays, increasing the contact area with the electrolyte and the NPs. This improved charge carrier extraction, generating 21.53 mA·cm^–2^ and a 3.45% photoconversion efficiency.^30^ Wei et al. implemented noble metal (Au, Ag, or Pt) nanoparticles and graphitic carbon nitride nanosheets on titania nanofibers, showing an improved photocatalytic activity for hydrogen evolution.^41^ Sn doping in ITO nanofibers affects the material’s morphology and electrical properties. Wu et al. showed that a Sn content in the range of 10–15 mol % resulted in minimum electrical resistivity for ITO nanofibers; hence, we used this composition range in the fibers prepared in this work.^38^

Electrospinning (ES) is an effective method to fabricate polymers, metals, and ceramics fibers with diameters ranging from nanometers to micrometer scales.^42−45^ In addition, electrospun nanofibers can be incorporated with nanoparticles either by in situ addition of the NPs to the electrospinning precursor or by a postspinning decoration of the fibrous mat.^46−48^ The final ceramic fiber phase is obtained after thermal treatment, which influences the morphology and material properties.^49,50^ Thus, when incorporating the NPs in situ, care must be taken to preserve their characteristics during heating. In this work, we embedded hematite nanoparticles into a fibrous ITO mat and measured the photoelectrochemical properties of this system We found a new route to disperse premade and well-defined 20–40 nm hematite NPs within the ITO scaffold while ensuring their photoactivity. Also, the new developed approach allows direct contact of each individual hematite NP with the conductive current collector. We are unaware of another work where hematite NPs were confined within a TCO electrospun nanofiber substrate. Furthermore, here, the NPs are in situ integrated during a facile and scalable synthesis process of the photoanode. The fibrous porous scaffold architecture demonstrated herein improved charge carrier transport and overall performance relative to its planar analog, despite a higher hematite content in the planar sample.

## 2 Experimental Section

### 2.1 Colloidal Synthesis of Hematite NPs

The NPs were synthesized by following an earlier report.^51^ Fe(acetylacetonate)_3_ (1 mmol, 0.353 g) and NaOH (3 mmol) were added into a mixed solvent of oleic acid (5 mL), ethanol (5 mL), and water (5 mL). The resulting mixture was transferred to a Teflon-lined stainless-steel autoclave (50 mL) and kept at 200 °C for 20 h. The products were collected by centrifugation at 4000 rpm for 3 min and dispersed in ∼2 mL of toluene, forming a weakly stable colloidal solution. Next, trimethylammonium-hydroxide (TMAOH) solution (0.5 mL, 2 M) was added to the toluene solution, resulting in immediate flocculation of the solution. The solution was centrifuged again at ∼3000 rpm for 1 min. The addition of doubly distilled water yielded a clear colloidal solution of hematite NPs, which was colloidally stable for at least several months. Flat photoelectrodes with different thicknesses (thin and thick) were prepared by spin coating of concentrated aqueous solution of hematite NPs on FTO-coated glass. To calculate the NPs concentration in solution and the film thickness from absorption, we used ε_450 nm_ = 250 M^–1^ cm^–1^ and ε_450 nm_ = 3.5 cm^–1^. Selected samples were treated in 5% H_2_ in N_2_ for 120 min at 450 °C to increase the conductivity of the sample.

### 2.2 ITO Nanofiber Synthesis

The electrospinning dry fiber components included PVP (*M*_w_ = 1,300,000), In(acetylacetonate)_3_, and SnCl_4_. The precursor solvents included a mixture of DMF, acetylacetone, and acetic acid. Precursor preparation is described in Table S1. An aqueous hematite NP suspension (20 mg·mL^–1^) that underwent the TMAOH treatment was added to the precursor solution above (Table S1) and stirred for 2 h. The precursor was loaded into a 5 mL syringe and placed horizontally facing a 15 cm-diameter cylindrical aluminum collector at a tip-to-collector distance of 15 cm. The syringe was attached to a high voltage supply (SL40P60; Spellman Hauppauge, New York, USA), and the collector was attached to a negative voltage supply (895025; Fluke). Operating conditions were +12 kV/–3 kV. A precursor feed rate of 0.3 mL·h^–1^ was maintained by a pump (KDS100; KD Scientific, Holliston, MA, USA). The relative humidity and temperature were maintained in the range of 35–40% and 20–25 °C, respectively. Approximately 50 μL of precursor solution was directly deposited onto a clean 1.5 × 3cm FTO-coated glass (10 Ω·sq^–1^, TEC T10, XOP Glass, Spain). FTO-coated glass samples were cleaned by sonicating in deionized (DI) water and detergent for 10 min, rinsing with DI water, sonicating in acetone for 10 min, rinsing with acetone, and sonicating in ethanol for 10 min. After this initial rinse, the samples were rinsed in ethanol, sonicated in 1 M KOH for 10 min, and finally sonicated in DI water for 20 min, rinsed with DI water, and dried under N_2_. The decorated fibrous layers were pressed onto the glass substrate to enhance adhesion by applying a pressure of 350 psi (0.5 metric ton) at a temperature of 170 °C (in the range of the glass transition temperature of the PVP polymer^49^) for 5 min. Then, the samples were placed in a tube furnace for multistage thermal treatment (Figure S1). Atomic layer deposition of TiO_2_ was applied by a hot-wall ALD reactor with ultrahigh-purity Ar gas as a carrier gas and purging between reactant exposures. The ALD-treated ITO nanofiber samples were prepared by introducing the samples to the hot reactor and dosing the reactant precursors into the Ar carrier gas. The sample temperature was 130 °C. The TiO_2_ layer was deposited by cycles of TiCl_4_–H_2_O (TiCl_4_ dose, 0.15 s; purge, 20 s; H_2_O dose, 0.25 s; purge, 60 s) using a Si thermal oxide wafer as a substrate. The thickness of each sample was measured by an ellipsometer. The sample was annealed in a tube furnace in air at 500 °C for 30 min. To increase the electrical conductivity, samples were treated in 5% H_2_ in N_2_ for 120 min at 450 °C.^52,53^

### 2.3 Materials Characterization

The samples were characterized by a high-resolution scanning electron microscope (HRSEM) (ULTRA Plus; Zeiss, Zurich, Switzerland). The phase compositions of the samples were characterized by X-ray diffraction (XRD) (MiniFlex; Rigaku, Japan). Phase identification was performed using a BEDE ZDS computer search/match program coupled with the ICDD (International Center for Diffraction Data) Powder Diffraction File database (2006). The mat pore structural properties were investigated by N_2_ physisorption at 77 K (3Flex apparatus, Micromeritics, GA, USA) after outgassing at 130 °C under high vacuum for 6 h. Thermal analysis and mass spectral (TGA/DTA-MS) measurements were measured at ambient pressure (Setsys Evolution 1750, Setaram, Caluire, France). The samples were heated from 25 to 850 °C at 5 °C·min^–1^ under an airflow of 20 mL·min^–1^. The TGA data were analyzed using Calisto Processing software (AKTS and Setaram). The transmittance (*T*) and reflectance (*R*) spectra of the photoanodes were measured using a PerkinElmer Lambda 950 UV/vis spectrometer from 1000 to 300 nm in an integrating sphere compartment to account for scattered light. The series resistance measurements were performed using a Zahner Zennium electrochemical workstation system in a three-electrode configuration using Ag/AgCl as a reference electrode and a Pt wire as a counter electrode. The measurement was performed by measuring the impedance of the system while applying a potential of 0.9 V_RHE_ with a 10 mV amplitude at a frequency of 10 kHz superimposed to it. Electrochemical impedance spectroscopy (EIS) was performed from 10 kHz to 0.3 Hz at bias potentials ranging from 1.13 to 2.03 V_RHE_. The potential was modulated using a 10 mV AC modulation around the baseline DC potential. X-ray photoelectron spectroscopy (XPS) was measured using an XPS Axis Supra (Kratos, UK) with a monochromatic Al Kα X-ray source. The band gap was evaluated by UV–visible spectral analysis using a UV–vis spectrophotometer (Lambda 1050, PerkinElmer).

### 2.4 Photoelectrochemical Testing

The photoelectrochemical performance of the samples was characterized using a “cappuccino cell” photoelectrochemical cell^54^ in a standard three-electrode setup in a 1 M NaOH aqueous solution with an aperture area of 0.11 cm^2^. A platinum wire coil was used as a counter electrode and a Hg/HgO/1 M NaOH electrode (RE-61AP, ALS-Japan) as a reference electrode. The potential was converted to the RHE scale using the Nernst equation. The measurements were carried out using a Compact Stat potentiostat (Ivium Technologies). Linear sweep voltammetry (LSV) measurements were carried out at a sweep rate of 10 mV/s. A broadband LED (Mightex GCS-6500-15-A0510, glacier white 6500 K, with main peaks at 450 and 550 nm as in Figure S2) was used as a light source. Measurements were conducted under a 100 mW·cm^–2^ illumination intensity. This light source was used for all the measurements under white light bias.

Incident photon-to-electron conversion efficiency (IPCE) measurements were performed by chronoamperometry measurements (using an Admiral Instruments SquidStat Prime potentiostat) at a potential of 1.53 V vs RHE while scanning the wavelength of incident monochromatic light with a Horiba 320iHR Xe lamp/monochromator system. The incident optical power was measured with a Newport 1936-R power meter with an 818-D8 calibrated Si detector, behind a 5 mm-diameter aperture matching the sample aperture in the cappuccino cell. The IPCE spectrum was calculated using the formula (*hc*/*e*λ) × (photocurrent/optical power), where *h* is Planck’s constant, *c* is the speed of light, *e* is the electronic charge, and λ is the wavelength. Collection and processing of the data are outlined in further detail in supplementary Figure S12.

## 3 Results

### 3.1 NP Synthesis and Characterization

**Figure 1:**
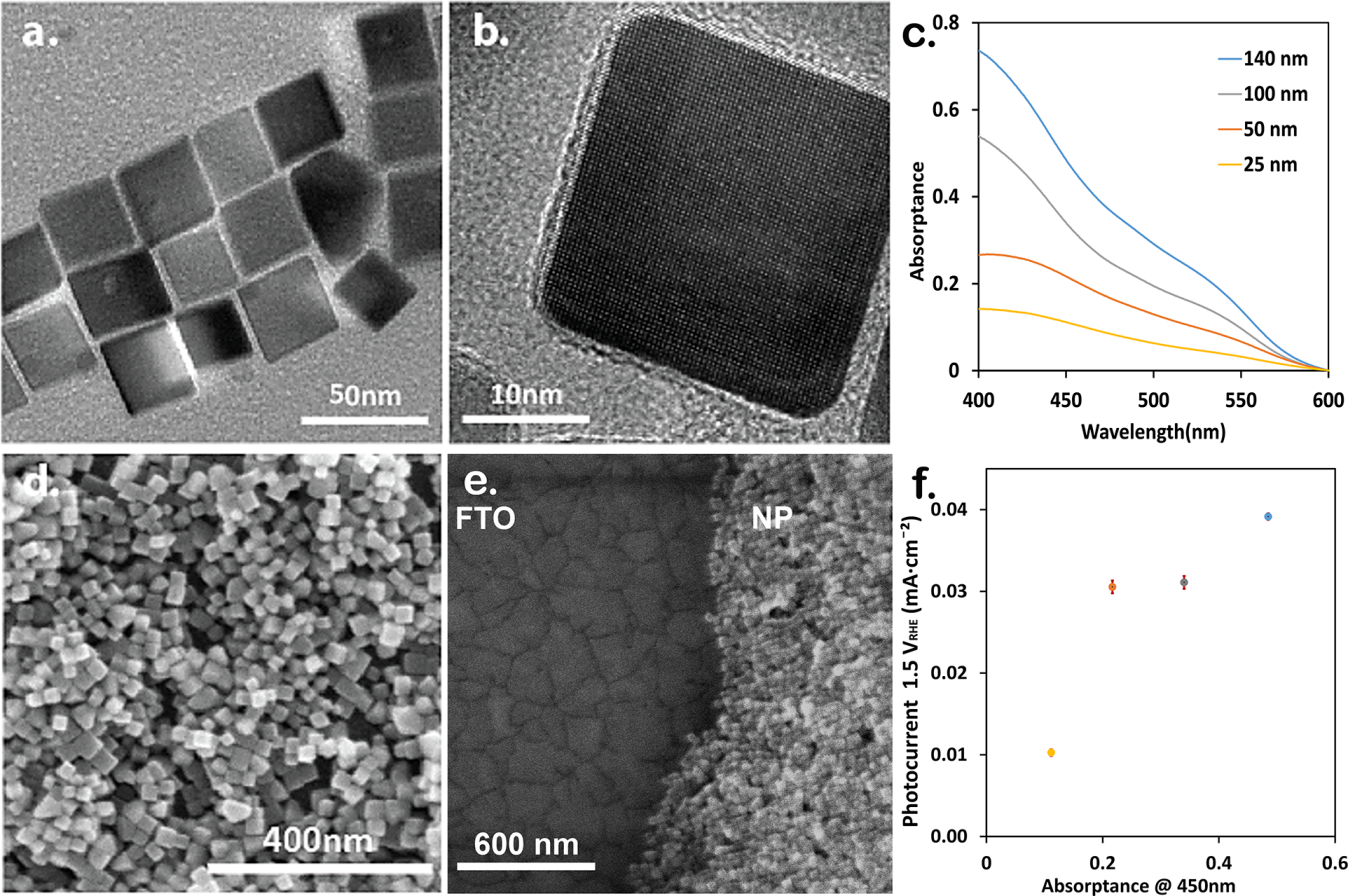
Hematite NC synthesis and planar layer preparation. (a,b) TEM images of hematite NPs. (c) Absorptance of planar photoelectrodes with different hematite NP coating thicknesses, as indicated in the legend. (d) SEM image of the layer surface (Abs._450 nm_ of ∼0.2, 50 nm), (e) tilt SEM image of the layer edge (Abs._450 nm_ of ∼0.2, 50 nm), and (f) average photocurrent at 1.5 V vs RHE as a function of the absorptance at 450 nm for the planar photoelectrodes shown in (c).

As described in the Experimental Section, hematite NPs were synthesized by a previously reported method.^51^Figure 1a presents the TEM images of these NPs, showing their quasi-cubic shape. This shape has its (012) faces exposed, as evidenced by the HRTEM image (Figure 1b). The size of the NPs is 26 ± 2 nm (Figure S3), close to the smallest size where the α-Fe_2_O_3_ phase is still stable in water; below this size, other phases of iron oxide become more stable.^19,20^ The amorphous frame of about 2 nm is due to the presence of oleic acid. This layer is detrimental to the PEC as it forms an insulating barrier to charge carrier separation and transfer. In addition, colloidal stability is crucial for photoelectrode fabrication, and the oleic acid-coated NPs are colloidally unstable. Hence, the NPs were transferred to water and coated with OH^–^ groups. The resulting NPs were colloidally stable in an aqueous solution with negligible scattering, as shown in the absorption spectra in Figure S3.

We used spin coating to prepare planar photoelectrodes with an NP layer of different thicknesses, using a highly concentrated solution of the hematite NPs to coat the FTO-coated glass substrates. The thickness of the planar photoelectrodes was controlled linearly by the solution concentration. Concentrations of 0.2, 0.15, 0.1, and 0.05 g/mL NPs in water resulted in Abs._450 nm_ of 0.1, 0.2, 0.35, and 0.5, respectively, as shown in Figure 1c. The features in the absorption spectra of the layer (red curve in Figure S3) are generally similar to those of the free NPs in solution (black curve in Figure S3), where the effect of scattering by the FTO substrate can explain the slight shifts. SEM images show the NP layer surface and its cross section (Figure 1d,e and Figure S3). From the absorption spectra, the average film thicknesses were calculated to be 140, 100, 50, and 25 nm, as plotted in Figure 1c. After PEC measurements, the hematite NPs preserved the same morphology and particle size as in Figure 1d,e.

As previously shown,^45^ treatment in a reducing environment can increase the electrical conductivity and charge transport, leading to the higher observed photocurrent. Untreated samples showed marginal or no photocurrent. The photocurrent measured at 1.5 V vs RHE as a function of absorbance at 450 nm is shown in Figure 1f. Generally, the photocurrent increases for a thicker hematite NP layer. However, the photocurrent enhancement decreases as thicker layers are deposited. The series resistance measured for a thick layer sample (Abs._450 nm_ of ∼0.5, 140 nm) was higher (415 Ω), while a thinner-layer sample (Abs._450 nm_ of ∼0.2, 50 nm) exhibited a lower series resistance (101 Ω). A possible reason for the observed results is poor charge transport of the top NPs, which absorb most of the light while being electrically disconnected from the current collector and do not contribute to the current. Thus, flat NP layers are limited by the amount of NPs in good electrical contact with the FTO current collector and enable efficient charge transport. To improve the charge transport from the hematite NPs to the TCO current collector and reduce the series resistance, we next studied the effect of integrating these NPs into porous electrodes as a strategy to achieve better performance.

### 3.2 Synthesis and Characterization of ITO Nanofibers Embedded with Hematite NPs

**Figure 2:**
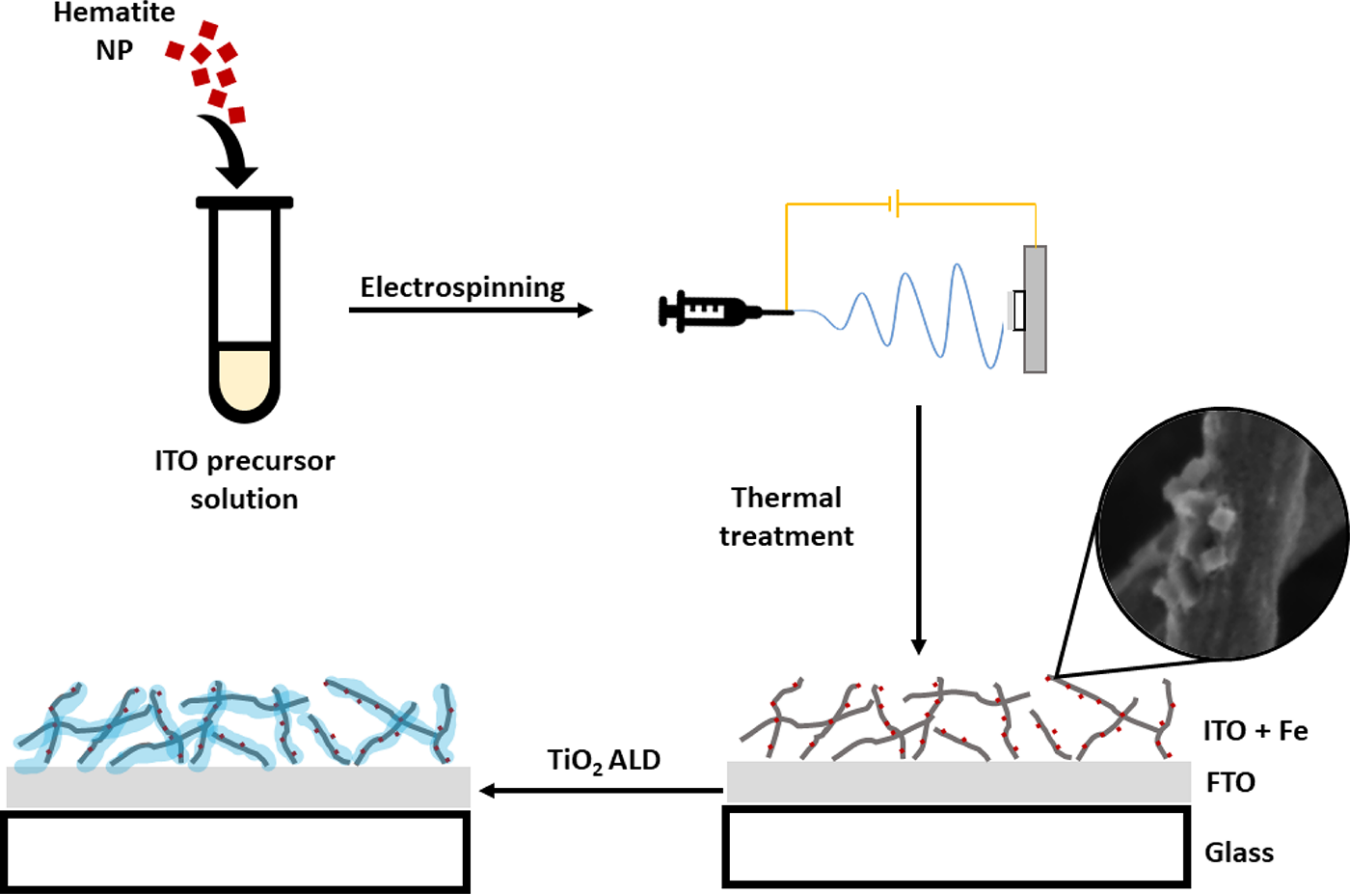
Schematic preparation procedure of ITO nanofibers embedded with hematite NPs on FTO-coated glass.

Hematite nanoparticles were embedded within the ITO fiber scaffolds during electrospinning (Figure 2). An aqueous hematite NP suspension was added to the electrospinning precursor solution (see Table S1). FTO-coated glass was attached directly to the counter electrode with conductive copper tape, and the NP-loaded ITO fibers were deposited on the FTO layer. The obtained ITO fibers were smooth, and the hematite NPs were spread unevenly within them (Figure S4a). The fibrous matrix consisted of nonwoven fibers (Figure S4b). The average diameter of the as-spun nanofibers was 110 ± 50 nm (Figure S4c).

After electrospinning, the sample was transferred to a furnace for thermal treatment. A stand-alone fibrous mat was analyzed in DTA/TGA-MS (Figure S5) to design the heating profile properly. An endothermic peak at 110 °C, due to evaporation of absorbed water, is followed by two exothermic peaks ending at ∼550 °C. The MS signal (Figure S5b) is well-matched with the TGA/DTA profile. During the first exothermic process at 240 °C, a significant amount of water, CO_2_, and some nitrogen compounds were released. These are associated with the oxidative decomposition of organic hydrocarbons and polymers. The second exothermic peak starts at 360 °C, accompanied mainly by a large amount of CO_2_ and carbon and a smaller amount of water and nitrogen species, indicating oxidation of carbon or carbon-rich organic residues. After the second exothermic event, no evolved gases were detected, indicating that the oxidation of organic residues was complete. A slow multistep heating profile (Figure S1) was used to burn out the organic parts and yield the oxide phase while ensuring the fibrous morphology and adhesion to the coated-glass substrate. The highest temperature during thermal treatment was 450 °C with a dwell time of 1 h to avoid damaging the FTO-coated glass, ensure conductivity of the FTO layer, and prevent ITO grain growth.^55^

**Figure 3:**
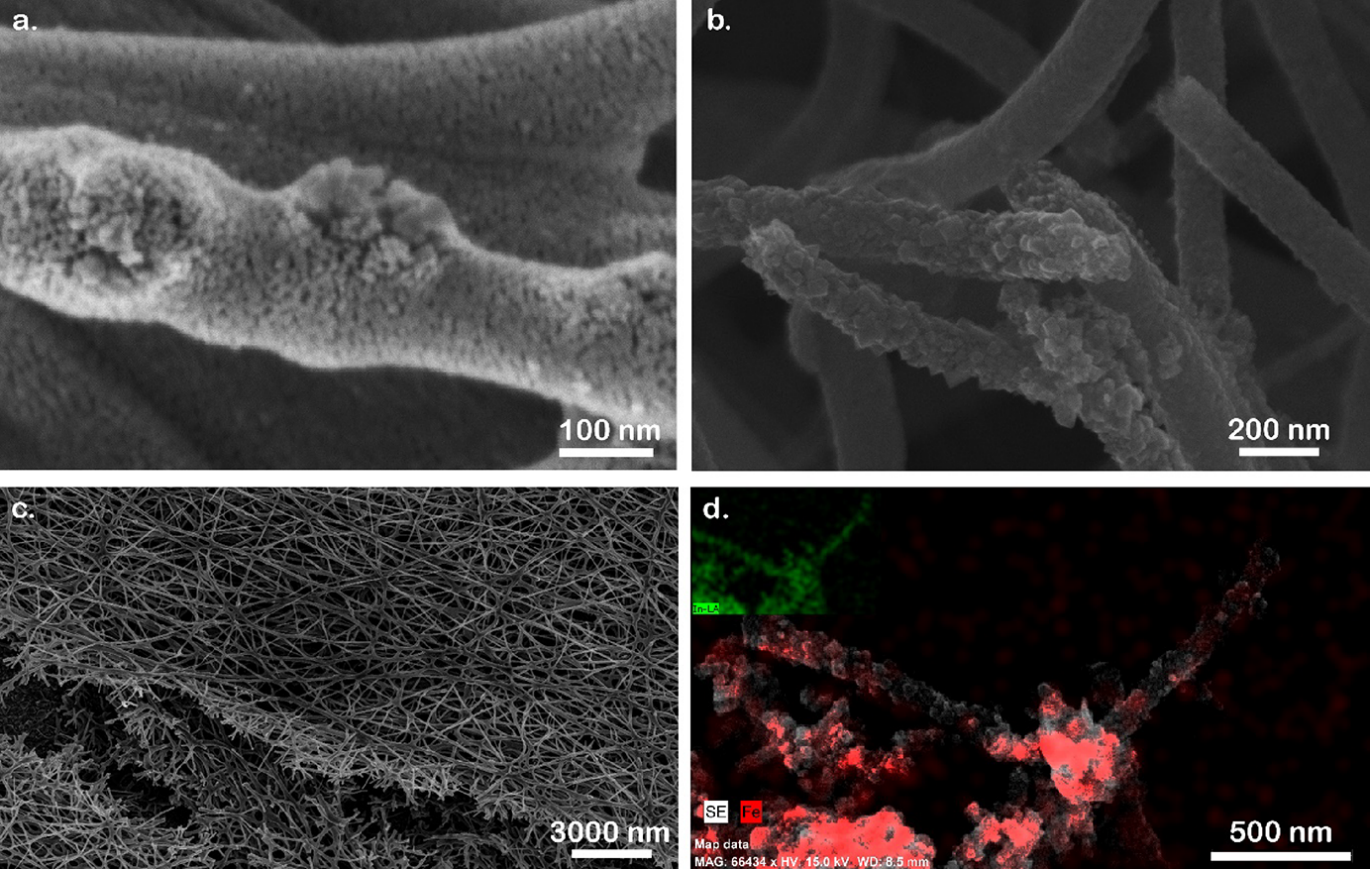
HRSEM of the nanoporous photoelectrode comprising ITO nanofibers with embedded hematite NPs deposited on FTO-coated glass at different magnifications. (a) 400,000×, (b) 100,000×, and (c) 10,000×, and (d) SEM-EDS Fe K-edge (red contrast) mapping of ITO nanofibers with hematite NPs. The inset shows an SEM-EDS In L-edge (green contrast) mapping of the same area.

After thermal treatment, XRD analysis was conducted on a stand-alone sample of only fibers, fibers deposited on FTO glass, and bare FTO glass (Figure S6a). XRD confirmed that the obtained fibers are of a cubic indium oxide phase. Tin oxide and hematite were not detected due to their small percentage relative to the dominant indium oxide phase in the stand-alone sample (only fibers). Bare FTO glass showed a tetragonal tin oxide phase. Fibers deposited on FTO glass showed the tin oxide phase (dominant) and the two major indium oxide phase peaks. Nitrogen physisorption measurements of the ITO nanofibers show a type IV isotherm (Figure S6b–d). The BET surface area, cumulative pore volume, and BJH average pore diameter were 61.5 ± 0.4 m^2^·g^–1^, 0.15 cm^3^·g^–1^, and 8.2 nm, respectively. After thermal treatment, the average fiber diameter was 85 ± 40 nm (Figure S7). In addition to linear shrinkage during thermal treatment, the diameter of the fibers decreases due to the decomposition of organic parts.^50^ The fiber morphology consists of the 20–40 nm hematite NP clusters surrounded by tiny ITO crystals and small pores (Figure 3a,b). The nonwoven fibrous structure of the mat is not destroyed by the thermal treatment (Figure 3c). EDS mapping confirmed that the hematite NPs are embedded into the ITO nanofibers (Figure 3d). In addition, we measured a series resistance of 85 Ω, lower than that of the planar photoelectrodes (100–450 Ω, depending on the layer thickness). The optical band gap of the ITO/hematite NP nanofibers was determined by the Tauc plot (Figure S8). The indirect band gap value was calculated by the baseline approach^56^ (Figure S8, red lines). The obtained band gap (3.5 eV) is lower than a previously reported value (3.65 eV) for deposited ITO layers with a thickness of 225 nm on a glass substrate.^57^ Other studies reported higher values (3.8–4.2 eV).^58^

**Figure 4:**
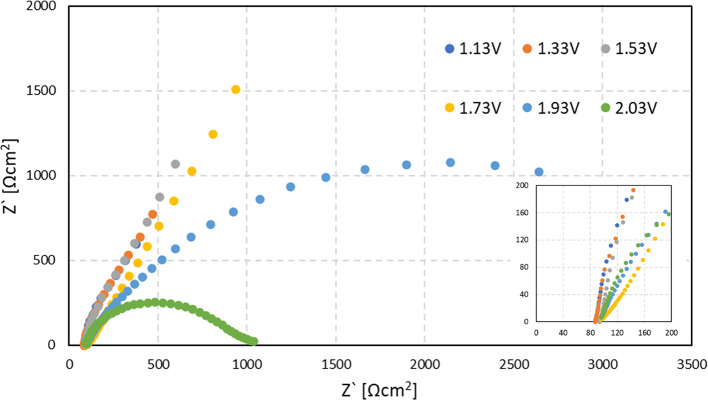
EIS measurements of the fibrous photoanode in the article in the dark. The measurements were performed under a fixed potential value range of 1.13–2.03 V_RHE_, as noted in the legend.

**Figure 5:**
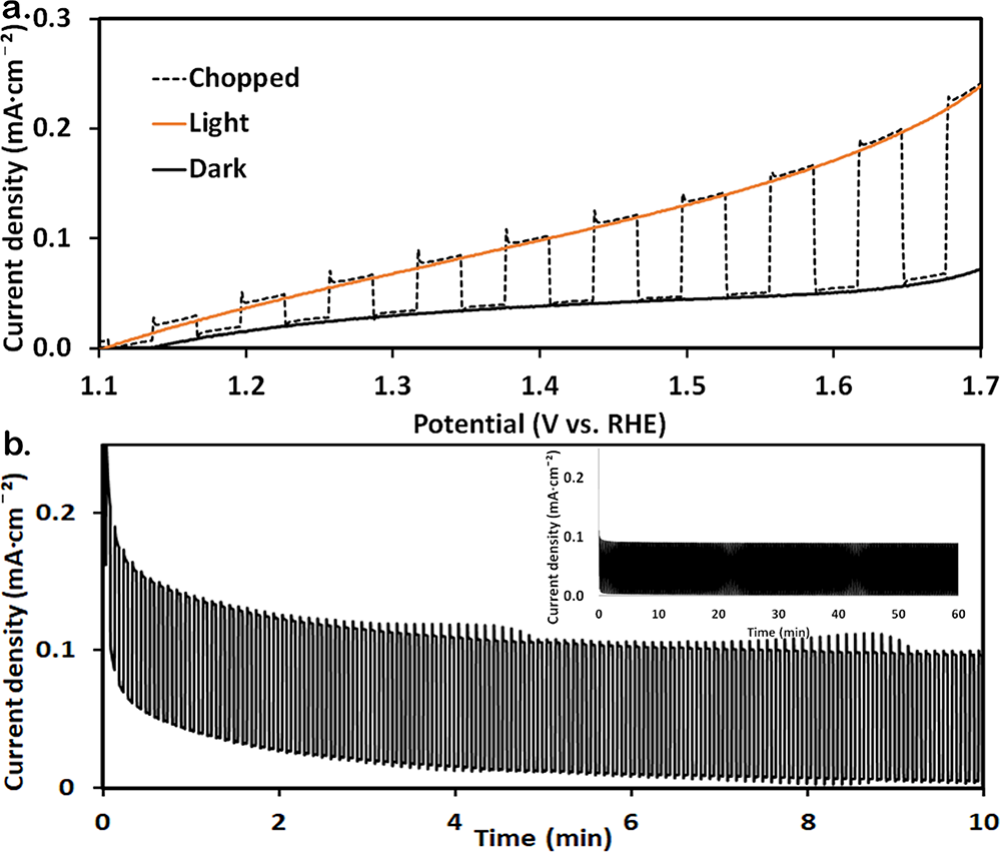
(a) Linear sweep voltammograms of a porous hematite photoanode measured in 1 M NaOH aqueous solution under dark, light, and chopped-light conditions before stability tests and (b) stability measurement under chopped light at a constant potential of 1.5 V_RHE_; inset: stability measurement for 1 h.

EIS measurements of the fibrous photoanode are shown in Figure 4. As seen from the high-frequency intercept with the *X*-axis (Figure 5, inset), the series resistance of the cell is ∼85 Ω. Only part of one semicircle can be observed in most of the measured potentials, representing high charge injection resistance from the photoanode to the electrolyte. The high resistance value is consistent with the negligible dark current measured in this range, as evident from the linear sweep voltammetry curves. In the case of the highest measured potential (2.03 V_RHE_), two definite semicircles are observed. The two semicircles can be attributed to processes of charge transfer from the bulk to the surface and charge injection to the electrolyte. Mott–Schottky (MS) analysis of the fibrous sample yielded a nonlinear step-like relationship between C^–2^ and the applied potential, indicating that MS analysis cannot be applied to this case.

### 3.3 Photoelectrochemical Measurements

The photoelectrochemical properties were examined by linear sweep voltammetry (LSV) under dark, light, and chopped-light illumination (see Figure 5a). Initially, samples showed a prominent redox wave (Figure S9a) related to the ITO redox activity at low potentials.^59,60^ To reduce the dark current, TiO_2_ was deposited by ALD on the fibers. A 2.5 nm-thick layer was sufficient to significantly decrease the dark current without drastically compromising PEC performance (Figure S9b). In potentiostatic measurements, the dark current of the ALD-TiO_2_-coated samples decayed to near-zero values after about 10 min, while the light current remained stable (Figure 5b). This was not the case for the uncoated samples, where a significant dark current was observed after 10 min (Figure S9c). This result shows the effectiveness of the ALD treatment in reducing the dark current without degrading the PEC performance. Embedding the hematite NPs into the fibrous matrix significantly improves the PEC performance (Figure 5a) relative to thick (Abs._450 nm_ of ∼0.5) and thin (Abs._450 nm_ of ∼0.2) planar photoanodes composed of the same hematite NPs (Figure 1). For example, at 1.5 V_RHE_, the photocurrent of the nanoporous photoanode with NP-loaded fibers was ∼0.1 mA·cm^–2^, whereas the best planar sample reached only ∼0.04 mA·cm^–2^. Stability measurement for 60 min was conducted under chopped light at a constant voltage of 1.5 V_RHE_ (Figure 4b, inset). The dark current decayed to near-zero values after 10 min (Figure 5b), whereas the photocurrent was stable (∼0.1 mA/cm^2^) throughout the full measurement (60 min).

Iandolo et al. deposited a 25 nm hematite film on an ITO substrate using physical vapor deposition and measured a photocurrent of ∼0.07 mA/cm^2^ at 1.5 V_RHE_.^61^ Hisatomi et al. observed a photocurrent below 0.1 mA/cm^2^ for a 9 nm hematite film on an FTO substrate without an underlayer.^62^ In addition, Singh et al. compared the performance of a hematite thin layer deposited on ITO and annealed at 350 or 500 °C. The photocurrents at annealing temperatures of 500 and 350 °C were 0.145 and 0.037 mA/cm^2^ at 1.23 V_RHE_, respectively. The authors suggested that at a higher annealing temperature of 500 °C, In can diffuse from the ITO substrate to the hematite, leading to better performance.^63^ Similarly, during the nanofiber production, the sample was exposed to a temperature of 500 °C, which can lead to diffusion of In to the hematite nanoparticles.

Ex situ HRSEM measurements showed that the morphology of the ITO nanofibers and hematite NPs was preserved after the PEC tests (Figure S10a,b). The surface chemical composition of the sample after testing was examined by XPS (Figure S11). XPS analysis showed that the Sn/(Sn + In) ratio was 14% (Table S2), within the optimal range.^38^ The XPS scan of the Fe 2p peak showed a peak centered at 716 eV (Figure S11a). The shift to a higher binding energy relative to the standard hematite characteristic peak (∼710 eV)^64,65^ indicates that the NPs possess a higher oxidation state after testing. Based on the XPS measurement, the observed Fe/In weight ratio is 10% (Table S2). After PEC testing, the ITO fibers and the ALD layer are reduced, indicating a possible electrochemical reduction of these components during testing. The Ti^4+^/Ti^3+^ ratio decreases after the reduction in H_2_ and furthermore after the PEC testing (Figure S11g). A larger number of Ti^3+^ species are associated with more defects and better conductivity of the TiO_2_ layer, resulting in a smaller activity due to leakage of the insulating layer (Figure S9c; the absorbed photocurrent before ALD is higher relative to that after ALD).^66,67^

## 4 Discussion

**Figure 6:**
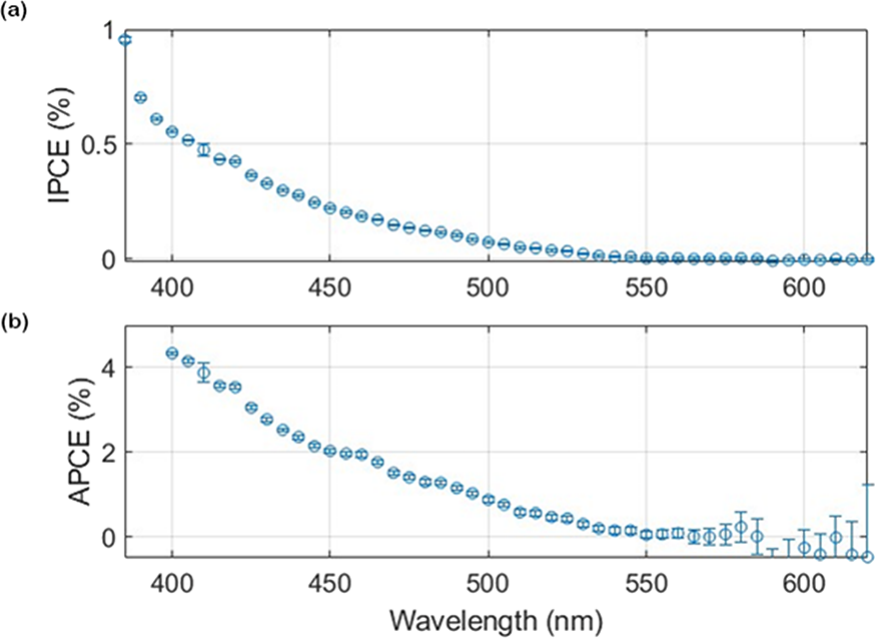
(a) IPCE spectrum measured at 0.6 V vs Hg/HgO (1.53 vs RHE) of the fibrous photoanode. (b) APCE recorded from the IPCE spectra of the fibrous photoanode.

We further characterize the photoanode by calculating the IPCE and APCE on a complementary fibrous sample. Figure 6a shows the calculated IPCE of the hematite NP ITO fibrous photoelectrode. Only the data above 380 nm are presented here since the incident power falls to zero (before recovering somewhat) at lower wavelengths. Refer to supplementary Figure S12 for more details on processing the time-dependent data. All the fine features in the photocurrent spectrum can be attributed to variations in the monochromator optical power, which results in an almost perfectly smooth and monotonic IPCE spectrum over the entire wavelength range after normalization. The onset of IPCE occurs near 540 nm, which is significantly below the typical onset in bulk hematite around 600 nm. We speculate that this could be related to the small size (∼30 nm from Figure 1a,b) of the hematite nanoparticles. Nevertheless, the onset of optical absorption from Figure 1c is 600 nm, consistent with prior works on hematite films and nanoparticles.^68,69^ Compared to the IPCE, this would suggest that the absorption above 540 nm is “unproductive”, which does not contribute to the photocurrent. Indeed, this behavior was observed in bulk hematite, showing significant “unproductive” absorption at wavelengths below 600 nm.^70^ By comparison, the present result shows a shift in IPCE, which may be owing to an additional size effect. Figure 6b shows the calculated APCE for each wavelength by dividing the IPCE by the net absorbance of hematite NPs (see below). The obtained IPCE and APCE values are within the range of previously reported samples of ultrathin hematite films with a thickness range of 12–50 nm (Table S3). It should be noted that here, the NPs are separated and confined within the scaffold matrix, where the hematite layer is continuous in the case of films. Further performance improvement could be achieved by doping and adding an underlayer (see Table S3).

**Figure 7:**
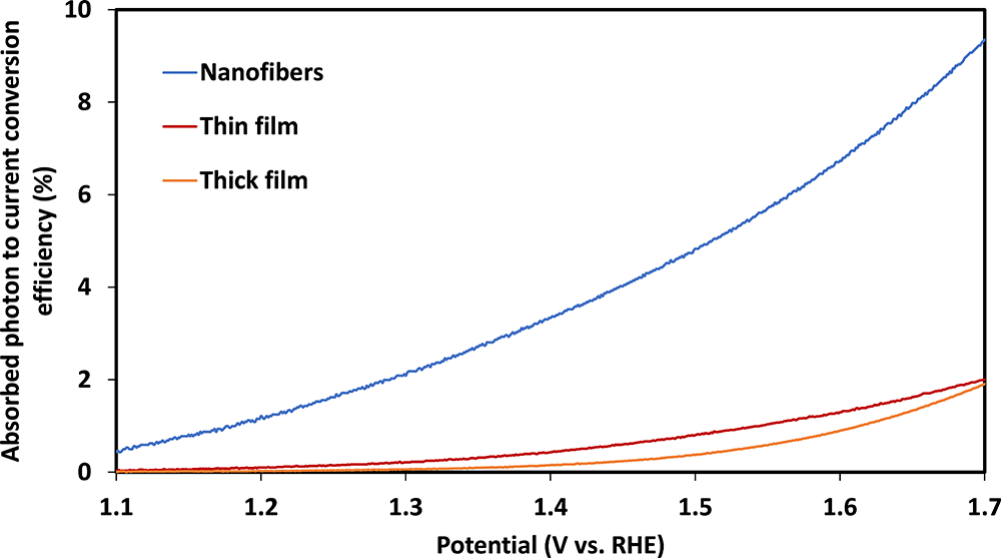
Absorbed photon-to-current conversion efficiency (integrated over the entire spectrum of absorbed light) as a function of the applied potential of fibrous, thin-layer (Abs._450 nm_ of ∼0.2, 50 nm) and thick-layer electrodes (Abs._450 nm_ of ∼0.5, 140 nm).

The improved PEC performance of the nanofiber sample compared to the thin- and thick-layer planar analogs is even more striking than the mere enhancement in the photocurrent because the amount of photocatalytic hematite NPs in the fibrous sample is lower than those in the planar counterparts (Table S2). We suggest that this significant effect arises from improved charge transport through the fibrous electrode. To further study this nontrivial effect, we compare the absorbed photon-to-current conversion efficiency (APCE, integrated over all the spectrum of light absorbed in the photoelectrode) of the nanofibers and planar electrodes (Figure 7). The photocurrent was normalized by accounting for the hematite NP adsorption. The reflectance data of the ITO nanofibers with hematite NPs are shown in Figure S12. The total absorbance includes contributions of the ITO nanofibers and hematite NPs. The effective absorbance of the hematite NPs was estimated similarly to a previous study.^58^ Since hematite does not absorb above 600 nm^13,71^ while ITO nanofibers absorb in the 400–800 nm range,^72,73^ we estimated the net absorbance of the hematite NPs by subtracting the background absorbance of ITO nanofibers that was fitted to the measured absorbance above 600 nm in the sample. Based on the net absorbance, we calculated the APCE integrated over the LED illumination wavelength range of 400–800 nm by dividing the photocurrent density by the absorbed photon current density within the hematite NPs. The obtained conversion efficiency was in the range of 5–10% at potentials above 1.5 V_RHE_, an order of magnitude higher than that of the planar analog electrode (at the same potential). A possible reason for the enhanced performance is better electrical conductivity and connectivity of the hematite NPs and current collector by embedding the hematite NPs within the ITO nanofiber scaffold. This is reflected by the lower series resistance, which is a crucial factor in PV and PEC devices. A high series resistance degrades the photoconversion efficiency by reducing the fill factor and maximum current of solar cell devices. These detrimental effects are diminished by embedding the hematite NPs within the ITO nanofibers, making them an integral part rather than separate components. This leads to the efficient integration of photocatalytic NPs in porous substrates for effective PEC water splitting devices. In addition, we postulate that the fractal structure in the fibrous photoanode enhances multiple scattering, giving rise to ray randomization and a longer path length within the layer, thereby enhancing the absorption.^74^

## 5 Conclusions

We demonstrated a scalable approach to integrate hematite NPs into a fibrous ITO scaffold with improved PEC performance relative to a planar electrode analog. The NPs were produced by solvothermal synthesis, resulting in a low PEC efficiency on flat electrodes. Hematite NPs embedded within the fibrous ITO scaffold showed a better current collection with a low series resistance and a significantly higher photocurrent and photoconversion efficiency. The higher APCE can be assigned to a better contact of the NPs to the substrate by embedding them with the ITO precursor solution. The ITO nanofibers and hematite NPs showed stable performance and maintained their morphology after the stability testing. The fibrous scaffold allows effective dispersion of the hematite NPs throughout the volume of the ITO scaffold and ensures effective charge collection. Further improvement may be achieved by doping the hematite NPs, and cocatalysts can be integrated into the fiber matrix to improve performance. This work provides new insights into the importance of good conductivity between the hematite NPs and the transparent current collector. It proposes a new and improved approach by embedding NPs in porous nanofiber scaffolds.

## Supporting Information Available

Electrospinning precursor, thermal treatment profile, LED emission spectrum, characterization of NPs and ITO nanofibers, XPS analysis, Tauc plots, characterization of ITO nanofibers with embedded hematite NPs, and IPCE and APCE supporting data (PDF)
