# Supplementary material for: Composite Indium Tin Oxide Nanofibers with Embedded Hematite Nanoparticles for Photoelectrochemical Water Splitting
Source: ACS Appl Mater Interfaces. 2022 Sep 12;14(37):41851–60. doi: 10.1021/acsami.2c05424 (PMC9501920; doi:10.1021/acsami.2c05424)
Supplement: Supplementary file 1 — am2c05424_si_001.pdf [file am2c05424_si_001.pdf]

Supporting Information

Composite indium tin oxide nanofibers with embedded hematite nanoparticles for photoelectrochemical water splitting

Oren Elishav[†,‡], David Stone[∥,‡], Anton Tsyganok[ǂ], Swetha Jayanthi[∥], David S. Ellis[ǂ], Tamir Yeshurun[†], Itzhak I. Maor[§], Adar Levi[∥], Vadim Beilin[§], Gennady E. Shter[§], Roie Yerushalmi[∥], Avner Rothschild[ǂ,†,], Uri Banin[∥,*], and Gideon S. Grader[§,†,*]*

† The Nancy & Stephen Grand Technion Energy Program (GTEP), Technion – Israel Institute of Technology, Haifa 3200002, Israel

∥ Institute of Chemistry and the Center for Nanoscience and Nanotechnology, The Hebrew University of Jerusalem, 91904, Jerusalem, Israel

ǂ Department of Materials Science and Engineering, Technion – Israel Institute of Technology, Haifa 3200002, Israel

§ The Wolfson Department of Chemical Engineering, Technion—Israel Institute of Technology, Haifa 3200003, Israel

† Faculty of Engineering, Tel Aviv University, Tel Aviv 6997801, Israel

Corresponding Author

* Email: avner@mt.technion.ac.il; uri.banin@mail.huji.ac.il; grader@technion.ac.il

Table S1. Electrospinning Precursor

**Component**	**wt.**
$SnCl_4$	1.30%
Acetylacetone	7.55%
PVP	11.11%
DMF	45.13%
$In(acetylacetonate)_3$	14.60%
Acetic acid	12.67%
Fe-NP suspension in water	7.63%

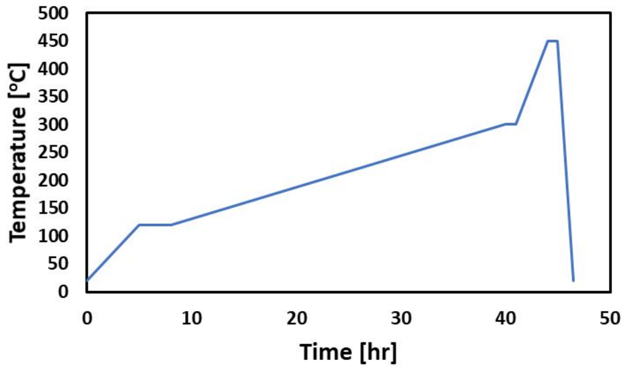

Figure S1. Thermal treatment profile in air.

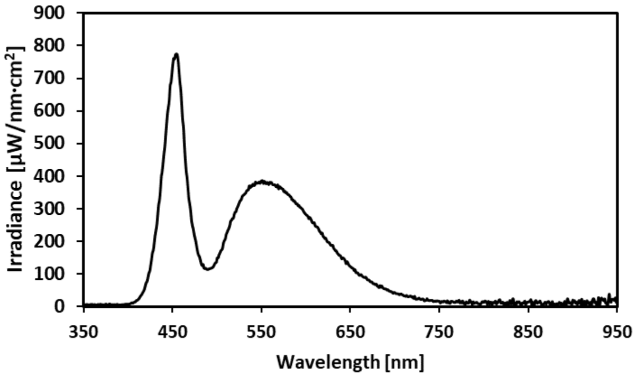

Figure S2. LED Emission spectrum measured before the experiment.

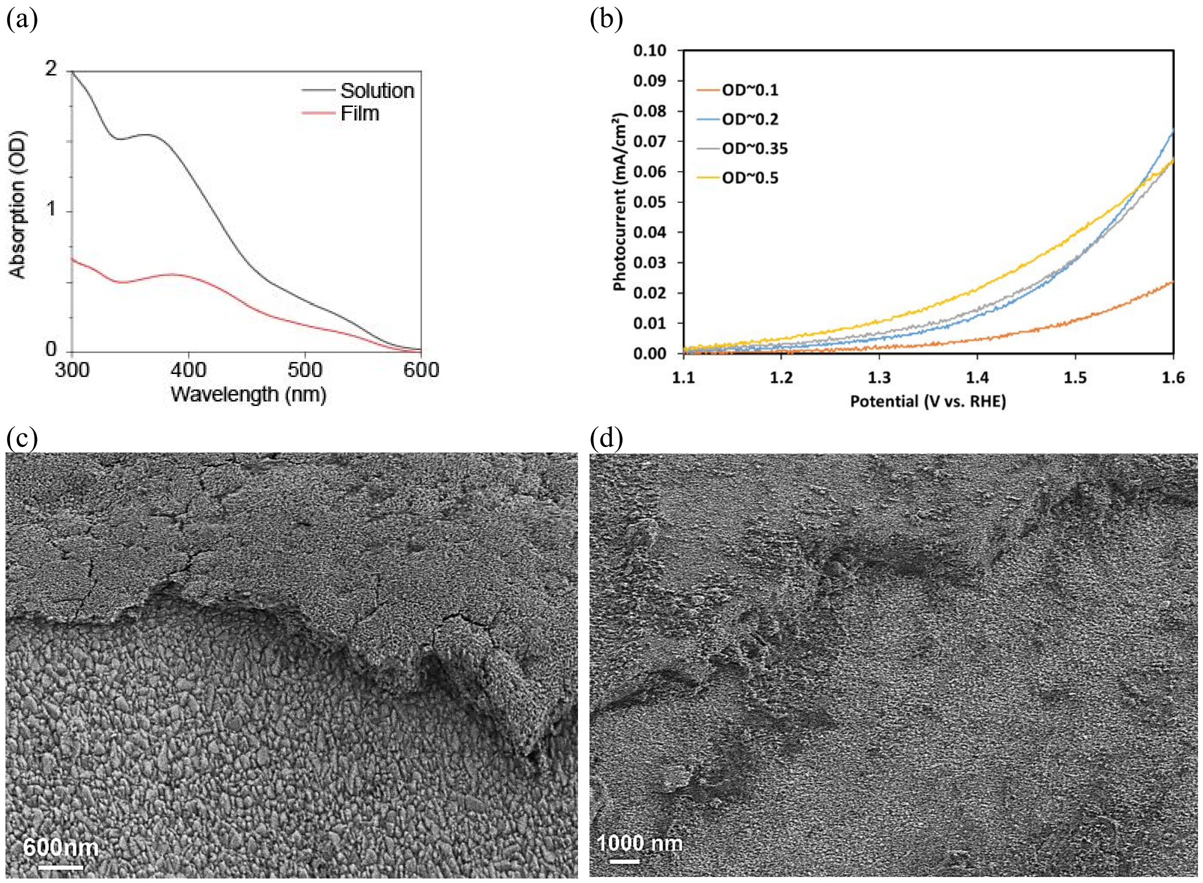

(e)

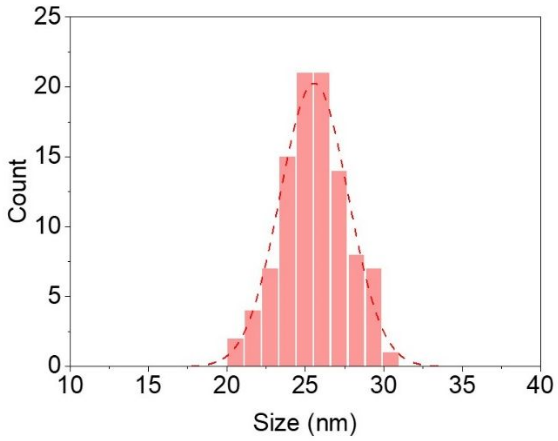

Figure S3. (a) Absorption of TMAOH-coated hematite NPs solution used for spin coating (diluted x1000) (black) and deposited as film on FTO glass (red), (b) Photocurrent measured in 1M NaOH aqueous solution for film samples with different thickness, (c) tilt SEM image of film edge with OD_{450nm}~0.1, (d) tilt SEM image of film edge with OD_{450nm}~0.35, and (e) NPs particles size distribution.

(a)

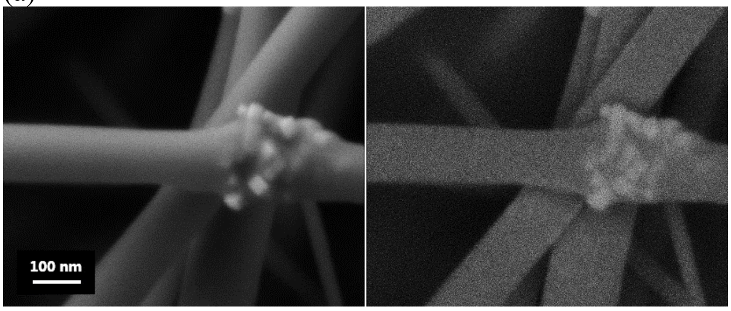

(b)

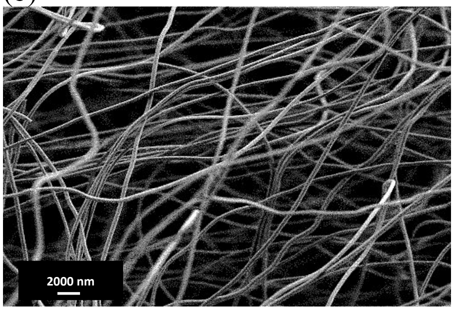

(c)

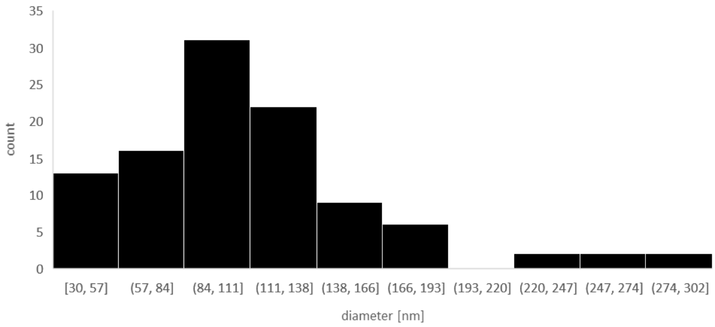

Figure S4. Characterization of ITO and hematite NP before thermal treatment (a) HRSEM image of nanofibers using secondary (left) and backscattered (right) detectors. (b) low magnification of nanofibers (c) diameter distribution

(a) (b)

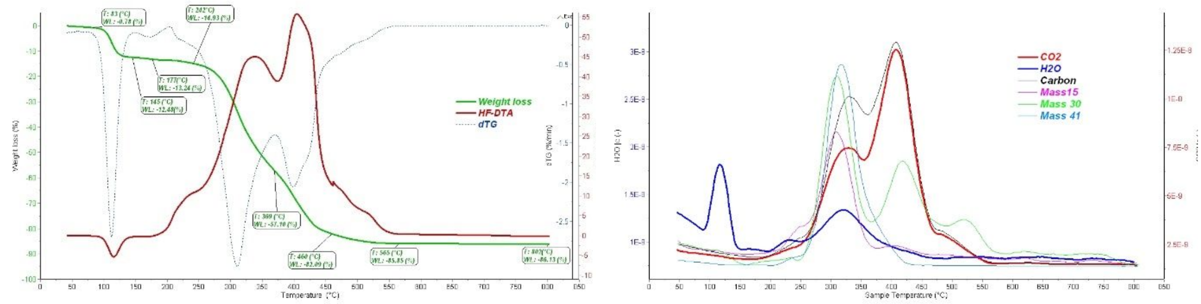

Figure S5. TGA/DTA-MS as-spun ITO nanofiber (a) thermal analysis, and (b) evolved gas mass analysis.

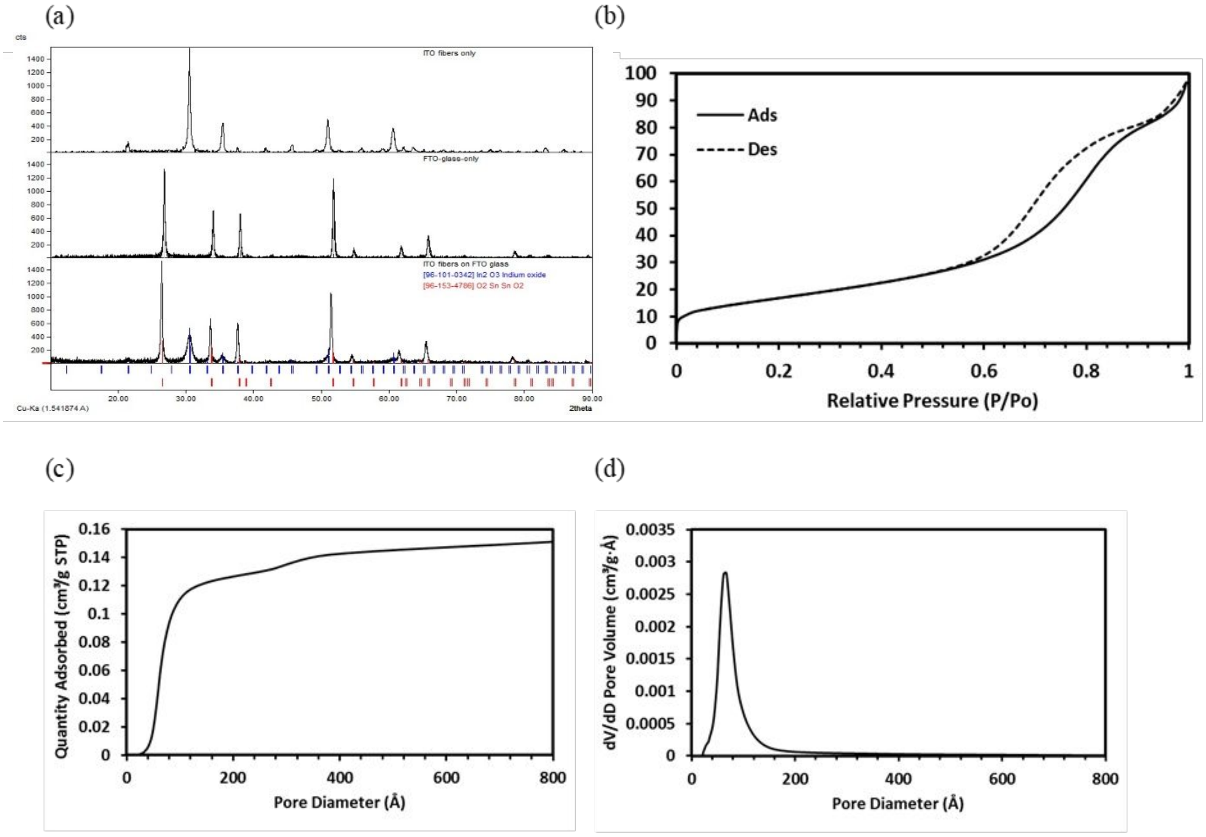

Figure S6. ITO Nanofibers' properties: (a)XRD patterns, (b)isotherm linear plot, (c) cumulative pores volume, and (d) pore volume distribution.

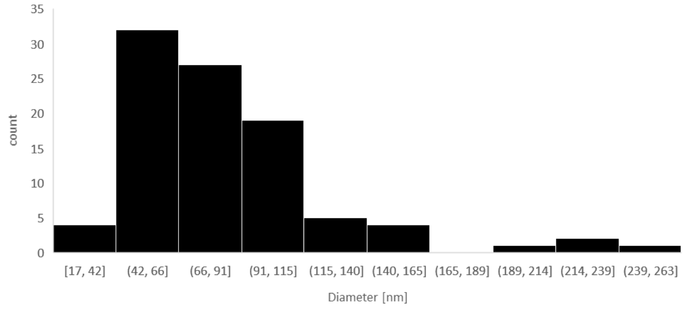

Figure S7. Diameter distribution after thermal treatment.

Table S2. XPS quantification elements percentage of film and fibrous electrodes

	Fibrous	Film -0.1 OD	Film -0.2 OD

	Atomic conc.	Err.	Atomic conc.	Err.	Atomic conc.	Err.
Fe 2p	1.1	0.1	2.9	0.13	8.19	0.14
Ti 2p	3.27	0.05	0	-	0	-
Sn 3d	0.89	0.02	3.76	0.04	7.98	0.09
O 1s	53.39	0.3	63.62	0.38	54.52	0.52
In 3d	5.43	0.04	0	-	0	-
C 1s	35.92	0.3	29.72	0.39	29.3	0.64
F 1s	0	-	0	-	0	-
**Fe/scaffold**[a]	**0.17**		**0.77**		**1.02**	

	**Film -0.35 OD**		**Film -0.5 OD**	
	Atomic conc.	Err.	Atomic conc.	Err.
Fe 2p	5.78	0.12	5.21	0.11
Ti 2p	0	-	0	-
Sn 3d	4.69	0.06	3.04	0.05
O 1s	65.64	0.45	54.53	0.39
In 3d	0	-	0	-
C 1s	23.9	0.49	37.22	0.41
F 1s	0	-	0	-
**Fe/scaffold**[a]	**1.23**		**1.71**	

[a] Scaffold= Sn+In+F

(a) (b)

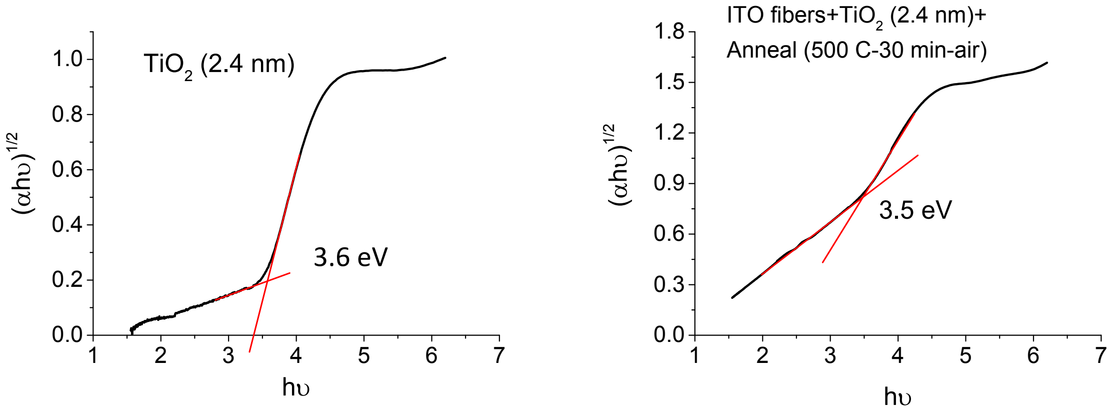

Figure S8. Tauc plot (a) TiO_2 deposited on quartz, and (b) ITO nanofibers with hematite NPs and TiO_2 ALD layer.

(a) (b)

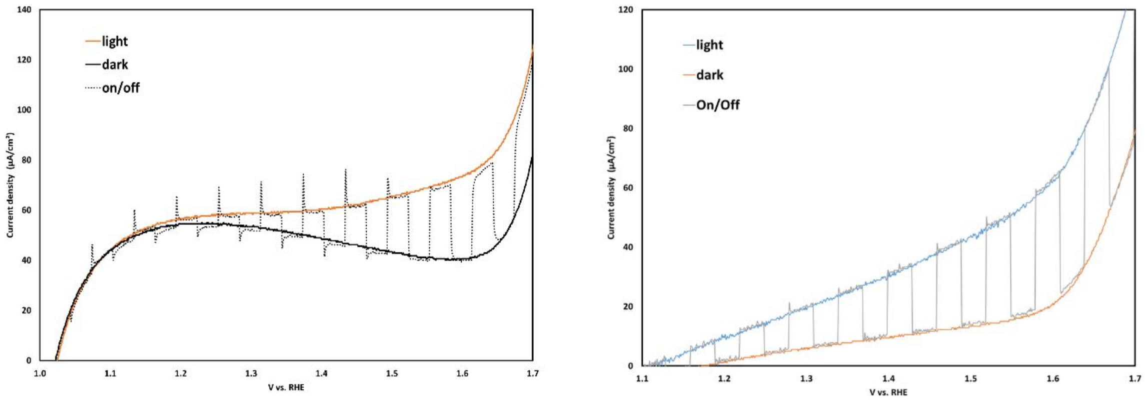

(c)

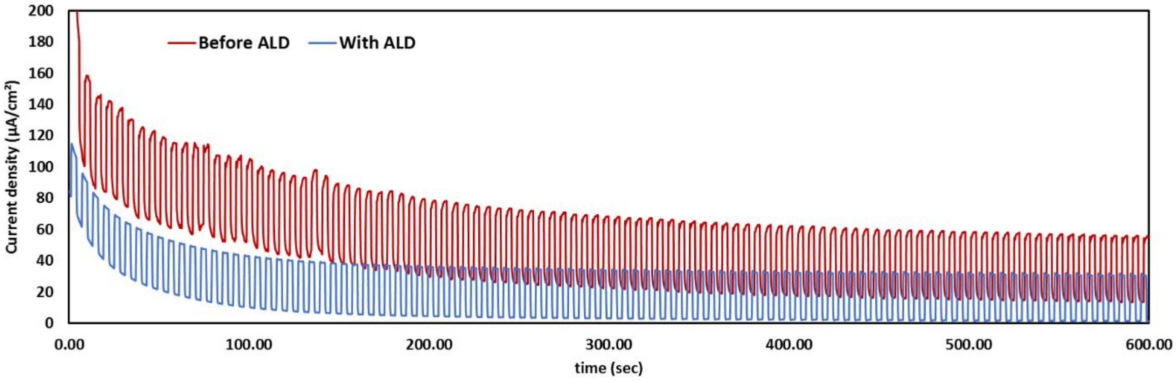

Figure S9. Linear sweep voltammograms measured in 1 M NaOH aqueous solution in the dark and under solar-simulated illumination. (a)before ALD, (b) after ALD 2.5 nm, and (c) stability measurements under chopped light amperometry at 1.5 V vs. RHE before and after (2.5nm) ALD.

(a)

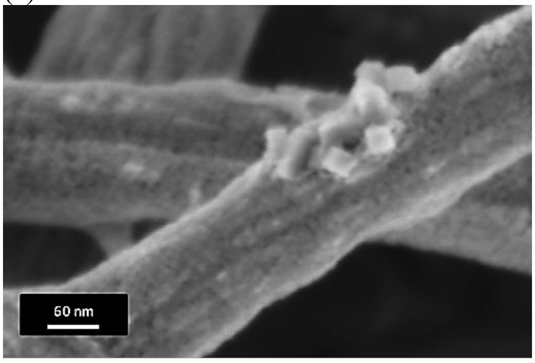

(b)

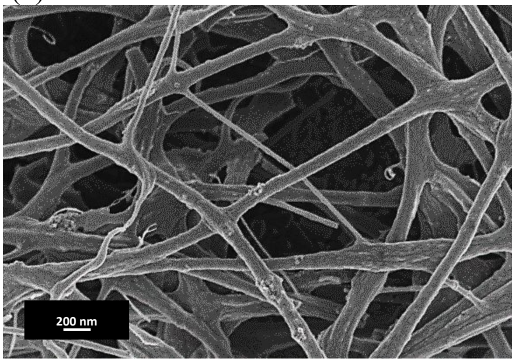

Figure S10. SEM image of ITO nanofibers with hematite NPs after PEC testing. (a) high magnification, and (b) low magnification

(a)

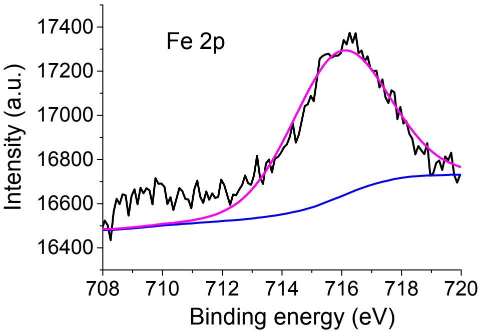

(b)

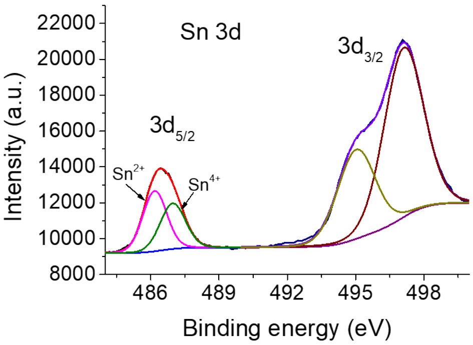

(c)

(d)

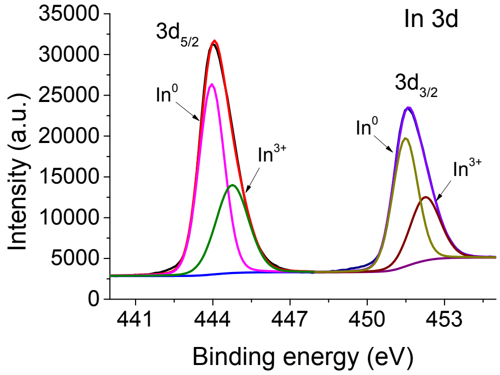

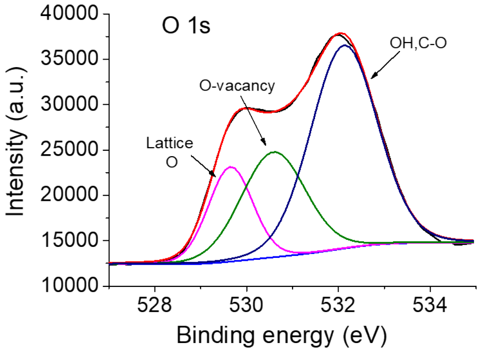

(e)

(f)

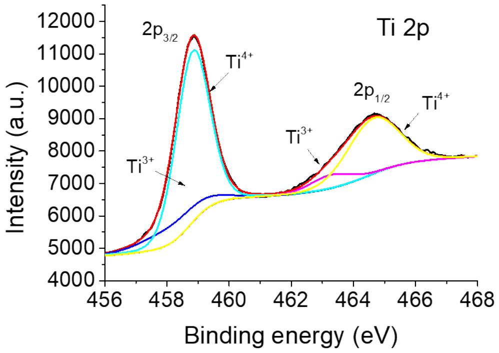

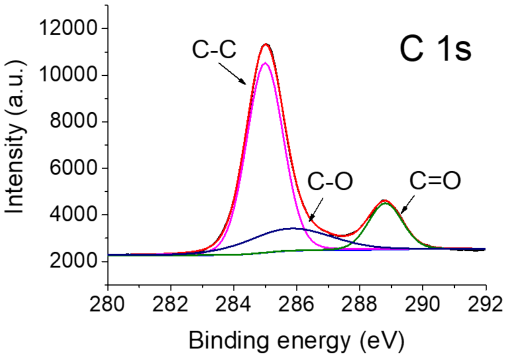

(g)

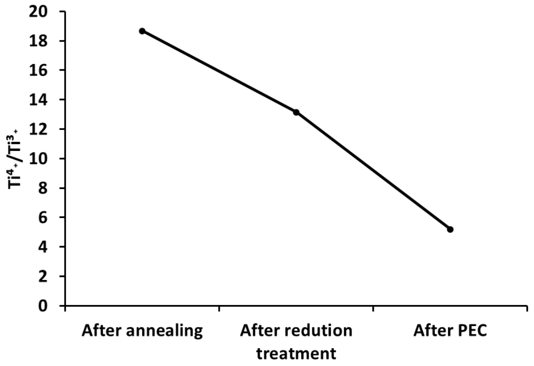

Figure S11. XPS of fibrous electrode (a) Fe 2p, (b) Sn 3d, (c) In 3d, (d) O 1s, (e) Ti 2p, (f) C 1s, and (g) Ti^{4+}/Ti^{3+} atomic ratio

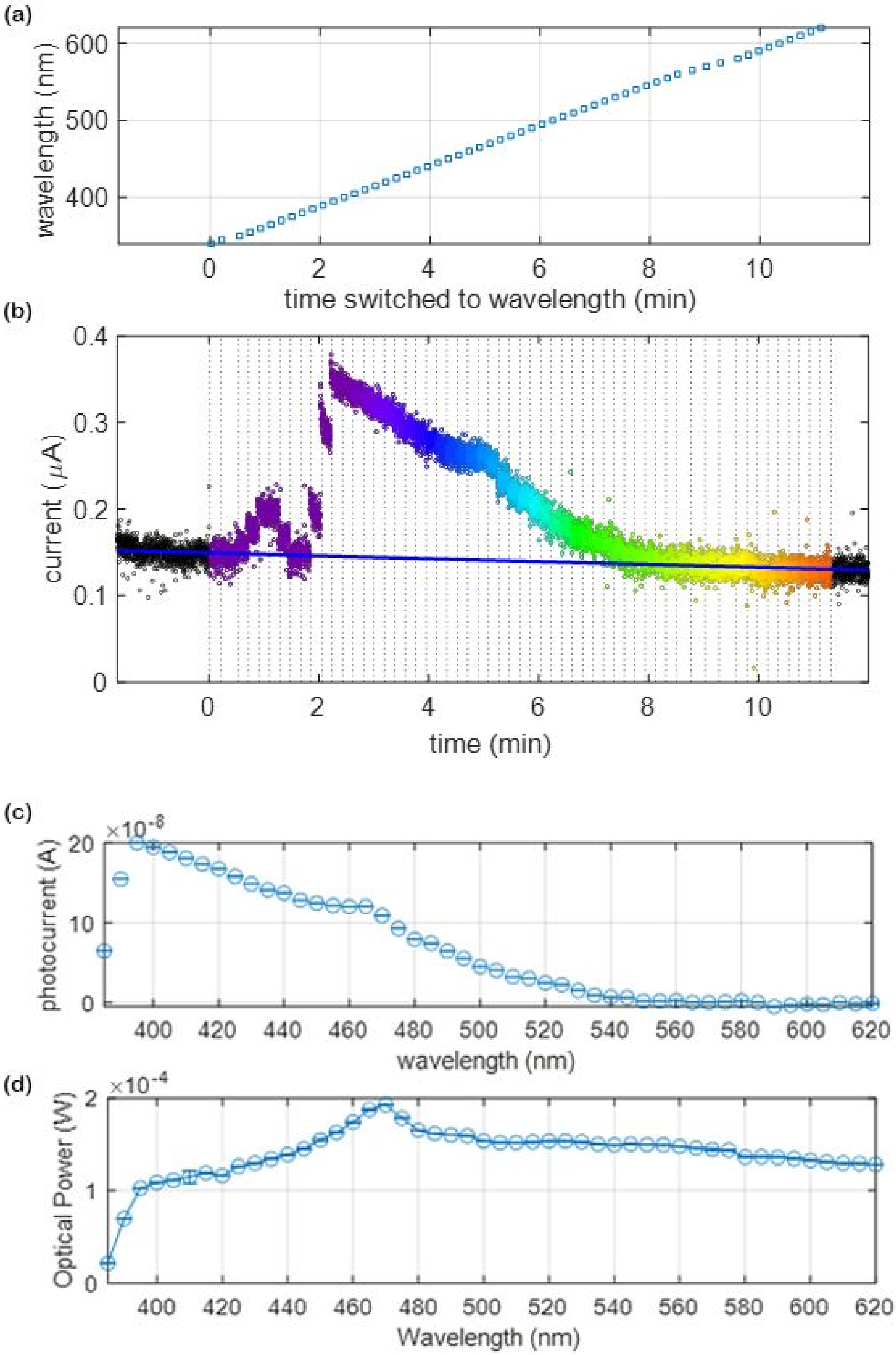

Figure S12. (a) Wavelength as a function of time during the monochromator scan, (b) Current as a function of time as the wavelength is scanned. The *t*=0 with respect to the monochromator scan is 3 minutes after the potential was applied. The colored part of the curve represents the time that the light source was scanning and incident on the sample (and indicates the wavelength, with violet used for the ultraviolet range), while the black part of the curve indicates no illumination on the sample. The vertical dashed lines correspond to the times of switching to each wavelength, with the last line being the estimated end of the scan before the light was shuttered. The dark current was estimated from a linear fit to the combined "dark" regions before and after the monochromator scan, shown in the figure: from *t*=-100 s to t=0, and from *t*=680 s to *t*=720s. The fit of this dark current is shown as a thick blue line. The photocurrent as a function of wavelength was calculated by first subtracting the latter dark current

from the total current, and then averaging this data within each of the constant wavelength regions (ie. between each of the vertical lines, (c) photocurrent, and (b) incident optical power

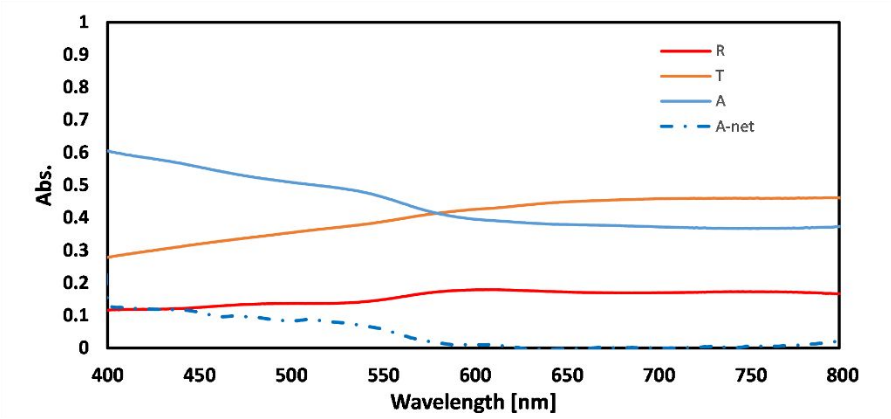

Figure S13. Absorptance (given by A = 1 − R – T) of FTO-coated glass deposited with ITO nanofibers and integrated with hematite NP. Where 'T', 'R', and 'A' stand for transmission, reflection, and absorption, respectively. 'A-net' stands for hematite NPs absorption only.

Table S3. Comparison of IPCE and APCE values of ultrathin hematite films

Sample	Thickness (nm)	λ (nm)	Voltage	IPCE	APCE	ref
This work	-	400	1.53 V vs. RHE	0.6%	4.2%	
Pure hematite	~47	400	1.23 V *vs.* RHE	0.1%	0.1%	1
Ti doped hematite	~47	400	1.23 V *vs.* RHE	5%	9.4%	1
SiO2 under-layered Ti doped hematite	~50	400	1.23 V *vs.* RHE	5.6%	13.67%	1
Pure hematite	14	455	1.23 V vs. RHE	0.8%	1.81%	2
bulk-Sn hematite	18.5	455	1.23 V vs. RHE	0.3%	0.67%	2
surf-Sn hematite	14	455	1.23 V vs. RHE	1.1%	2.57%	2
top-Sn hematite	16.8	455	1.23 V vs. RHE	3.1%	6.76%	2
2 at. % Ti-doped hematite films	21	400	0.6 V vs Ag/AgCl	2.41 %	-	3
2 at. % Ti-doped hematite films	29	400	0.6 V vs Ag/AgCl	2.56%	-	3
2 at. % Ti-doped hematite films	50	400	0.6 V vs Ag/AgCl	3.96%	-	3
ALD- hematite	30	440	190 mV vs SCE	5.8%	15%	4
SiOx underlayer hematite	23.3	400	1.43 V vs. RHE	-	15%	5

SiOx underlayer hematite	12.5	400	1.43 V vs. RHE	-	18.67%	[5]
ATO/hematite	50-80	400	1.5 V vs. RHE	7-15%	-	[6]

References

(1) Kang, M. J.; Kang, Y. S. Ultrathin Insulating Under-Layer with a Hematite Thin Film for Enhanced Photoelectrochemical (PEC) Water Splitting Activity. *J. Mater. Chem. A* **2015**, *3* (30), 15723–15728. https://doi.org/10.1039/C5TA03468J.

(2) Hufnagel, A. G.; Hajiyani, H.; Zhang, S.; Li, T.; Kasian, O.; Gault, B.; Breitbach, B.; Bein, T.; Fattakhova-Rohlfing, D.; Scheu, C.; et al. Why Tin-Doping Enhances the Efficiency of Hematite Photoanodes for Water Splitting—The Full Picture. *Adv. Funct. Mater.* **2018**, *28* (52), 1804472. https://doi.org/10.1002/ADFM.201804472.

(3) Rioult, M.; Magnan, H.; Stanescu, D.; Barbier, A. Single Crystalline Hematite Films for Solar Water Splitting: Ti-Doping and Thickness Effects. *J. Phys. Chem. C* **2014**, *118* (6), 3007–3014. https://doi.org/10.1021/JP500290J/ASSET/IMAGES/LARGE/JP-2014-00290J_0008.JPEG.

(4) Klahr, B. M.; Martinson, A. B. F.; Hamann, T. W. Photoelectrochemical Investigation of Ultrathin Film Iron Oxide Solar Cells Prepared by Atomic Layer Deposition. *Langmuir* **2011**, *27* (1), 461–468. https://doi.org/10.1021/LA103541N/SUPPL_FILE/LA103541N_SI_001.PDF.

(5) Formal, F. Le; Grätzel, M.; Sivula, K. Controlling Photoactivity in Ultrathin Hematite Films for Solar Water-Splitting. *Adv. Funct. Mater.* **2010**, *20* (7), 1099–1107. https://doi.org/10.1002/ADFM.200902060.

(6) Wang, L.; Palacios-Padrós, A.; Kirchgeorg, R.; Tighineanu, A.; Schmuki, P. Enhanced Photoelectrochemical Water Splitting Efficiency of a Hematite–Ordered Sb:SnO2 Host–Guest System. *ChemSusChem* **2014**, *7* (2), 421–424. https://doi.org/10.1002/CSSC.201301120.
